# Validity of ICD‐9 and ICD‐10 codes used to identify acute liver injury: A study in three European data sources

**DOI:** 10.1002/pds.4803

**Published:** 2019-06-06

**Authors:** Joan Forns, Miguel Cainzos‐Achirica, Maja Hellfritzsch, Rosa Morros, Beatriz Poblador‐Plou, Jesper Hallas, Maria Giner‐Soriano, Alexandra Prados‐Torres, Anton Pottegård, Jordi Cortés, Jordi Castellsagué, Emmanuelle Jacquot, Nicolas Deltour, Susana Perez‐Gutthann, Manel Pladevall

**Affiliations:** ^1^ Epidemiology RTI Health Solutions Barcelona Spain; ^2^ Clinical Pharmacology and Pharmacy, Department of Public Health University of Southern Denmark Odense Denmark; ^3^ Fundació Institut Universitari per a la recerca a l'Atenció Primària de Salut Jordi Gol i Gurina (IDIAPJGol) Barcelona Spain; ^4^ Universitat Autònoma de Barcelona, Bellaterra (Cerdanyola del Vallès) Barcelona Spain; ^5^ Institut Català de la Salut Barcelona Spain; ^6^ EpiChron Research Group. Aragon Health Sciences Institute (IACS), IIS Aragón, REDISSEC ISCIII Zaragoza Spain; ^7^ Departament d'Estadística i Investigació Operativa Universitat Politècnica de Catalunya Barcelona Spain; ^8^ Pharmacoepidemiology Department Les Laboratoires Servier Paris France; ^9^ The Center for Health Policy and Health Services Research, Henry Ford Health System Detroit MI USA

**Keywords:** acute liver injury, antidepressants, pharmacoepidemiology, validation

## Abstract

**Purpose:**

Validating cases of acute liver injury (ALI) in health care data sources is challenging. Previous validation studies reported low positive predictive values (PPVs).

**Methods:**

Case validation was undertaken in a study conducted from 2009 to 2014 assessing the risk of ALI in antidepressants users in databases in Spain (EpiChron and SIDIAP) and the Danish National Health Registers. Three ALI definitions were evaluated: primary (specific hospital discharge codes), secondary (specific and nonspecific hospital discharge codes), and tertiary (specific and nonspecific hospital and outpatient codes). The validation included review of patient profiles (EpiChron and SIDIAP) and of clinical data from medical records (EpiChron and Denmark). ALI cases were confirmed when liver enzyme values met a definition by an international working group.

**Results:**

Overall PPVs (95% CIs) for the study ALI definitions were, for the primary ALI definition, 84% (60%‐97%) (EpiChron), 60% (26%‐88%) (SIDIAP), and 74% (60%‐85%) (Denmark); for the secondary ALI definition, 65% (45%‐81%) (EpiChron), 40% (19%‐64%) (SIDIAP), and 70% (64%‐77%) (Denmark); and for the tertiary ALI definition, 25% (18%‐34%) (EpiChron), 8% (7%‐9%) (SIDIAP), and 47% (42%‐52%) (Denmark). The overall PPVs were higher for specific than for nonspecific codes and for hospital discharge than for outpatient codes. The nonspecific code “unspecified jaundice” had high PPVs in Denmark.

**Conclusions:**

PPVs obtained apply to patients using antidepressants without preexisting liver disease or ALI risk factors. To maximize validity, studies on ALI should prioritize hospital specific discharge codes and should include hospital codes for unspecified jaundice. Case validation is required when ALI outpatient cases are considered.

**KEY POINTS**

- Case validation of acute liver injury (ALI) was conducted in two Spanish databases, EpiChron and SIDIAP, and in the Danish national registers.
- Validation of potential cases included patient profiles review and adjudication based on clinical data extracted from medical records.
- The overall PPVs obtained were higher for specific than for nonspecific codes and for hospital discharge than for outpatient codes.
- The nonspecific code “unspecified jaundice” had high PPVs for all ALI definitions in Denmark but not in the Spanish databases.
- To maximize validity, studies on ALI should prioritize hospital specific discharge codes.

## 1 INTRODUCTION

Acute liver injury (ALI) is defined as a sudden appearance of liver test abnormalities and includes a broad spectrum of clinical scenarios, ranging from mild abnormal biochemical liver values to acute liver failure.^1^, ^2^

Previous validation studies have shown that identification of potential ALI events through diagnosis and procedural codes is challenging and that most validated algorithms have positive predictive values (PPVs) below 60%,^3^, ^4^, ^5^ except in one study, which reported PPVs >75%.^6^ All previous studies highlight the need for validation by medical record review when conducting studies of ALI based on automated health care data sources. This is especially important in drug safety studies, in which reliance on algorithms alone for automated case identification will most likely result in misclassification and overestimation of the true incidence of ALI and biased effect estimates.

As part of a recent post‐authorization safety study (PASS) conducted in five European data sources investigating the potential risk of ALI associated with the use of agomelatine and nine other antidepressant drugs,^7^ validation of the algorithms used to identify ALI cases was conducted. This was done via medical record review in three of those data sources: two Spanish health care databases and the Danish National Health Registers.

## 2 METHODS

The objective of this study was to determine the ability of three ALI definitions to correctly identify ALI cases in three automated health care data sources. Specifically, we aimed to validate the following:

- An ALI definition including only main hospital discharge diagnosis specific codes
- An ALI definition including main hospital discharge diagnosis specific and nonspecific codes
- An ALI definition including main hospital discharge and also outpatient diagnosis codes (both specific and nonspecific)

### 2.1 Study setting

Five automated health care databases were used in the agomelatine PASS.^7^ Three of these were used to conduct a validation study: in Spain, the EpiChron Cohort Study from Aragon Health Sciences Institute (Aragón, Spain)^8^ and the Information System for Research in Primary Care (SIDIAP) (Catalonia, Spain)^9^; and in Denmark, the Danish National Health Registers (Denmark).^10^, ^11^ The main characteristics of each database are included in Supplementary eTable S1. Of the two databases that were not used, validation by review of medical records is not an option in the German Pharmacoepidemiological Research Database (GePaRD) (Germany)^12^, ^13^, ^14^ and was not feasible within the study timeframe in the Swedish National Registers (Sweden).^15^, ^16^ Nevertheless, an external validation study was conducted in Germany,^17^ the results of which will be presented in a separate publication.

### 2.2 Identification and definition of ALI

Cases of ALI were identified in cohorts of new users of the 10 study antidepressants evaluated in the agomelatine PASS study between 2009 and 2014^7^: citalopram, agomelatine, fluoxetine, paroxetine, sertraline, escitalopram, duloxetine, venlafaxine, mirtazapine, and amitriptyline. Individuals aged 18 years or older at the date of their first‐recorded prescription fill of any of the study antidepressants during the study period(s) entered the cohort if they (a) had not received a prescription fill for the same study antidepressant within the prior 12 months (new users) and (b) had at least 12 months of continuous enrolment in the data source before the first prescription fill. Absence of pregnancy at the start date of antidepressant use was an additional inclusion criterion for women. Patients with a history of liver disease or risk factors for liver disease (eg, alcohol and drug abuse and dependence‐related disorders), chronic biliary or pancreatic disease, malignancy, or other life‐threatening conditions (eg, HIV infection) were excluded from the study cohort (Supplementary eMethods).

**Figure 1:**
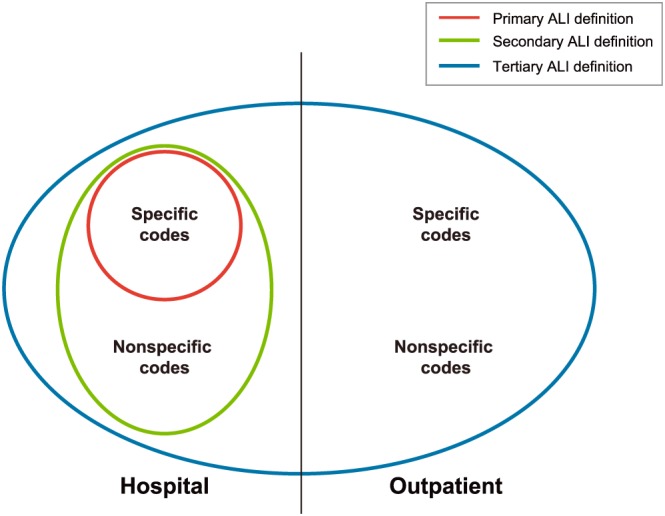
Definition of the study ALI definition algorithms^a^ ^a^ALI definition refers to the case‐identifying algorithms only. By definition, the secondary ALI definition in the analysis included only cases confirmed after validation [Colour figure can be viewed at wileyonlinelibrary.com]

Three algorithms corresponding to three ALI definitions were used in the agomelatine PASS to automatically identify potential ALI cases based on diagnosis codes (Table 1).^7^, ^18^ These definitions include combinations of codes that have shown higher (specific) or lower (nonspecific) PPVs in previous validation studies.^3^, ^4^, ^5^, ^6^ The primary ALI definition was defined as any patient with a *specific* main hospital discharge diagnosis code of ALI from either the *International Classification of Diseases, Ninth Revision, Clinical Modification* (ICD‐9‐CM) or the *International Statistical Classification of Diseases and Related Health Problems, Tenth Revision* (ICD‐100) (Table 2). The primary ALI definition was not validated per se, but the specific codes identifying the primary ALI definition were included in the secondary ALI definition, which underwent validation. The algorithm used to identify potential cases of the secondary study ALI definition was defined as any patient with a main hospital *specific* or *nonspecific* discharge code (ICD‐9‐CM or ICD‐10) for ALI. Finally, the algorithm for the tertiary ALI definition was assessed using specific and nonspecific codes from either ICD‐9‐CM or ICD‐10 identified in both hospital and outpatient settings. In EpiChron, International Classification of Primary Care (ICPC) codes were used to identify outpatient cases of the tertiary ALI definition and ICD‐9‐CM to identify hospital cases. In SIDIAP, ICD‐10‐CM was used to identify primary care diagnoses and ICD‐9‐CM to identify hospital cases. In Denmark, primary care codes were not available, and therefore only hospital ICD‐10 codes were used both for case identification and to apply exclusion criteria. The interplay between the three ALI definitions is displayed in Figure 1.

**Table 1:** ICD‐9‐CM and ICD‐10 codes relevant to acute liver injury

Code	Description
Specific codes	
ICD‐9‐CM	
570.x	Acute and subacute necrosis of liver
572.2	Hepatic coma
573.3	Hepatitis unspecified
ICD‐10	
K71.0	Toxic liver disease with cholestasis
K71.1	Toxic liver disease with hepatic necrosis
K71.2	Toxic liver disease with acute hepatitis
K71.6	Toxic liver disease with hepatitis, not elsewhere classified
K71.9	Toxic liver disease, unspecified
K72.0	Acute and subacute hepatic failure
K72.9	Hepatic failure, unspecified
K75.9	Inflammatory liver disease, unspecified
K76.2	Central hemorrhagic necrosis of liver
Nonspecific codes	
ICD‐9‐CM	
573.8	Other specified disorders of liver
573.9	Unspecified disorders of liver
782.4	Jaundice, unspecified, not of newborn
V42.7	Liver transplant
790.4	Nonspecific elevation of transaminase or lactic acid dehydrogenase
789.1	Hepatomegaly
ICD‐10	
K76.8	Other specified diseases of liver
K76.9	Liver disease, unspecified
R17	Unspecified jaundice, excludes neonatal
R16.0	Hepatomegaly, not elsewhere classified
R16.2	Hepatomegaly with splenomegaly, not elsewhere classified
R74.0	Nonspecific elevation of transaminase and lactic acid dehydrogenase
Z94.4	Liver transplant
ICPC	
D97	Liver disease (specified or unspecified)
D13	Jaundice
D23	Hepatomegaly
A91	Abnormal results investigations

Abbreviations: ICD‐9‐CM, *International Classification of Diseases, Ninth Revision, Clinical Modification*; ICD‐10, *International Statistical Classification of Diseases and Related Health Problems, Tenth Revision*; ICPC, International Classification of Primary Care.

**Table 2:** Positive predictive values (PPVs) of study ALI definitions and of overall specific and nonspecific codes used to identify potential acute liver injury (ALI) cases (nonevaluable cases not included)

	EpiChron			SIDIAP			Denmark		
	Total^a^	TP	PPV, % (95% CI)^b^	Total^a^	TP	PPV, % (95% CI)^b^	Total^a^	TP	PPV, % (95% CI)^b^
Secondary ALI definition	31	20	64.5 (45.4‐80.8)	20	8	40.0 (19.1‐63.9)	213	150	70.4 (63.8‐76.5)
Specific codes^c^	19	16	84.2 (60.4‐96.6)	10	6	60.0 (26.2‐87.8)	50	37	74.0 (59.7‐85.4)
Nonspecific codes	12	4	33.3 (9.9‐65.1)	10	2	20.0 (2.5‐55.6)	163	113	69.3 (61.6‐76.3)
Tertiary ALI definition	134	34	25.4 (18.3‐33.6)	2,242	172	7.7 (6.6‐8.9)	443	208	47.0 (42.2‐51.7)
Specific codes	18	15	83.3 (58.6‐96.4)	46	16	34.8 (21.4‐50.2)	73	50	68.5 (56.6‐78.9)
Nonspecific codes	116	19	16.4 (10.2‐24.4)	2,196	156	7.1 (6.1‐8.3)	370	158	42.7 (37.6‐47.9)

Abbreviations: CI, confidence interval; SIDIAP, Information System for Research in Primary Care; TP, true positives.

a Total of evaluable cases. Nonevaluable cases for the secondary and tertiary ALI definitions were 9 and 104 in EpiChron, 14 and 584 in SIDIAP, and 28 and 66 in Denmark.

b PPV was calculated as PPV = confirmed cases / (true positives + false positives). Results are presented as positive predictive values (%) and their 95% CIs.

c Equivalent to the PPVs for the study primary ALI definition (specific hospital discharge codes).

### 2.3 Diagnostic criteria for ALI

Potential cases of ALI identified with the electronic algorithms and reviewed by adjudicators were considered confirmed (true positives)^19^ if any of the following three qualifying criteria for increases in serum levels with <1 year of persistence were met (aspartate transaminase [AST] levels could be used instead of ALT levels only if ALT levels were unavailable and there was no known muscle pathology driving the rise in AST):

- ≥ 5 x upper limit of normal (ULN) alanine aminotransferase (ALT)
- ≥ 2 x ULN alkaline phosphatase (ALP)
- ≥ 3 x ULN ALT and > 2 x ULN bilirubin
  The requirement of less than 1 year of persistence of the liver function test abnormalities was introduced to ensure that cases had ALI and not chronic liver injury.^19^ This criterion was evaluated using the most recent liver enzymes results from the period 12 to 24 months before the index date to check whether they were not elevated beyond 10% of the ULN (if no results were available, the criterion was considered as met).

A *false‐positive case* of ALI was defined as a potential case with enough data to be evaluated but that did not meet the criteria to be classified as a confirmed case of ALI. A *nonevaluable case* of ALI was defined as a potential case that lacked some of the required liver enzyme results to be evaluated.

### 2.4 Validation steps

The strategy for validating potential cases identified by automated algorithms across the three data sources included up to three steps: review of patient profiles (which is a deidentified chronological listing of medical events and drug prescriptions and is used to detect exclusion diagnoses missed by the electronic algorithm and to provide an initial assignment of case status), medical record abstraction of relevant clinical data by trained health care professionals, and review of abstracted data and case adjudication by trained physicians. However, local adaptations were required in Denmark and SIDIAP to reflect data availability and/or local regulations (Supplementary eTable S2). In Denmark, patient profiles were not reviewed due to the very limited clinical information available. Also, primary care data were not available. Finally, patients with study exclusion criteria not identified by hospital codes were excluded during either the abstraction or the review of the abstracted information from medical records. In SIDIAP, source hospital medical records were not accessible; therefore, patient profile review relied only on liver enzyme results available from primary care and yielded the final case classifications in this database. Cases were reviewed both by trained physicians for all secondary ALI definition potential cases and by an electronic algorithm for the tertiary ALI definition due to the large number of identified potential cases.

Several quality control checks and measures were performed. All the health care professionals at each site involved in the validation, including nurses, clinical pharmacists and physicians, received training on the validation processes. In EpiChron, for quality control purposes, patient profiles of a random sample of 10 potential cases were reviewed independently by a second physician and a random sample of 25% of the confirmed cases and of 10 inpatient nonevaluable cases also were reviewed by a second physician. In SIDIAP, for the tertiary ALI definition, an electronic algorithm evaluated all potential cases, and 10% of them were also evaluated manually by trained professionals blinded to the study exposure. A very high level of agreement (kappa statistic equal to or larger than 0.95) between the algorithm and the manual reviewers was obtained before the algorithm was generalized; agreement between the two clinician reviewers was also assessed (kappa statistic = 1). Similarly, in Denmark, an algorithm was created to evaluate potential cases. Trained physicians manually reviewed 50 potential cases, all of which were also reviewed using the automated algorithm. All potential cases were evaluated using the automated algorithm only after the kappa measuring the agreement between manual review and the algorithm reached 1.

### 2.5 Statistical analyses

Validity of the electronic algorithms and individual codes used to identify potential cases of ALI for the secondary and tertiary ALI definitions were assessed by calculating the overall PPV of the algorithm, the overall PPVs of the specific and nonspecific codes, and the PPV of each individual code. PPVs for the primary ALI definition were indirectly calculated through the specific codes of the secondary ALI definition. The PPV was calculated as true positives / (true positives + false positives). In a sensitivity analysis, nonevaluable cases were included in the PPV denominator.

The PPVs were computed with 95% confidence intervals (CIs) for binomial proportions by the exact method using Stata software^20^—version 12 at EpiChron and version 14 at Denmark. At SIDIAP, SAS statistical software (version 9.4; SAS Institute, Inc; Cary, North Carolina) and R software version 3.3.1 were used.

## 3 RESULTS

**Figure 2:**
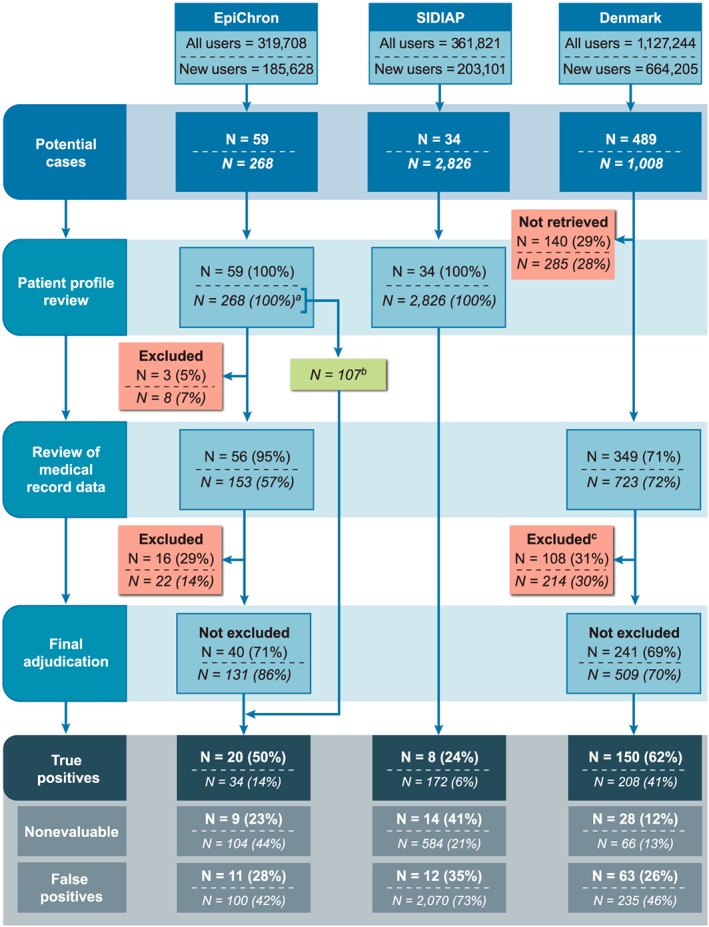
Flowchart with the flow of potential cases through the case validation process: Secondary (regular font) and tertiary (italics) ALI definitions
Note: In each cell, the first number refers to secondary ALI definitions, and the second number refers to tertiary ALI definitions.^a^ One hundred fifteen patients did not undergo further validation due to the lack of additional hospital data for those cases. Among them, eight patients were excluded based on the presence of exclusion or censoring criteria and did not undergo further validation.^b^ One hundred seven patients identified on ambulatory codes and with lack of additional hospital data were directly adjudicated during the patient profile phase. Among them, three were classified as true positives, 69 as false positives, and 35 were considered nonevaluable.^c^ Patients with study exclusion criteria not identified by hospital codes were excluded during the abstraction or review of medical records. [Colour figure can be viewed at wileyonlinelibrary.com]

The number of users of antidepressants and the final number of new users (after applying inclusion/exclusion criteria) in the three databases in which validation of potential cases was conducted are included Supplementary eTable S3. In EpiChron, SIDIAP, and Denmark, 59, 34, and 489 potential cases of the secondary ALI definition, respectively, were identified; and 268, 2826, and 1008 potential cases of the tertiary ALI definition were identified. Then, 31, 20, and 213 potential cases of the secondary ALI definition were considered evaluable cases; and 134, 2242, and 443 potential cases of the tertiary ALI definition were considered evaluable cases. Of them, 20, 8, and 150 cases of the secondary ALI definition and 34, 172, and 208 cases of the tertiary ALI definition were confirmed (true positives) after validation (Figure 2).

Regarding the tertiary ALI definition, which includes the total number of cases for all ALI definitions (see Figure 1), more than 70% of true positives in Denmark and SIDIAP and 56% of true positives in EpiChron were females. Overall, the age group with the highest number of true positives was patients 80 years and older, followed by patients aged 50 to 79 years (Supplementary eTable S4).

The overall PPVs for the algorithm used to identify potential cases of the secondary ALI definition were 65% (95% CI, 45%‐81%) in EpiChron, 40% (95% CI, 19%‐64%) in SIDIAP, and 70% (95% CI, 64%‐77%) in Denmark (Table 2). As discussed in the Methods section, the primary ALI definition was indirectly validated through the specific hospital discharge codes used in the secondary ALI definition, for which the overall PPVs were 84% (95% CI, 60%‐97%) in EpiChron, 60% (95% CI, 26%‐88%) in SIDIAP, and 74% (95% CI, 60%‐85%) in Denmark. The overall PPVs for the specific codes were higher than those for the nonspecific codes in all data sources (Table 2). In EpiChron and SIDIAP, the individual specific code 570.x (acute and subacute necrosis of liver) had the highest PPV, while the code 573.3 (hepatitis unspecified) captured the highest proportion of true positives (Table 3). In Denmark, the individual specific codes K71.2 (toxic liver disease with acute hepatitis) and K71.6 (toxic liver disease with hepatitis, not elsewhere specified) obtained the highest PPVs and captured the highest proportion of true positives (Table 4). None of the nonspecific codes captured more than two true positives in EpiChron and SIDIAP (Table 3). Conversely, in Denmark, the individual nonspecific code R17 (unspecified jaundice, excludes neonatal) contributed the largest number of true positives and had the highest PPV among all individual specific or nonspecific hospital discharge codes.

**Table 3:** Positive predictive values (PPVs) of specific and nonspecific codes used to identify potential acute liver injury (ALI) cases: Secondary (regular font) and tertiary (italics) ALI definitions in data sources using ICD‐9‐CM codes (nonevaluable cases not included)

	EpiChron			SIDIAP		
	Total	TP	PPV, % (95% CI)^a^	Total	TP	PPV, % (95% CI)^a^
Specific codes						
570.x Acute and subacute necrosis of liver						
Secondary ALI definition	5	5	100.0 (47.82‐100.0)	3	3	100.0 (29.2‐100.0)
*Tertiary ALI definition*	*5*	*5*	*100.0 (47.8*‐*100.0)*	*1*	*1*	*100.0 (2.5*‐*100.0)*
572.2 Hepatic coma						
Secondary ALI definition	1	0	0.0 (0.0‐97.5)	0	‐	‐
*Tertiary ALI definition*	*1*	*0*	*0 (0*‐*97.5)*	*0*	*‐*	*‐*
573.3 Hepatitis unspecified						
Secondary ALI definition	13	11	84.6 (54.6‐98.1)	7	3	42.9 (9.9‐81.6)
*Tertiary ALI definition*	*12*	*10*	*83.3 (51.6*‐*97.9)*	*4*	*3*	*75.0 (19.4*‐*99.4)*
Nonspecific codes						
573.8 Other specified disorders of liver						
Secondary ALI definition	9	2	22.2 (2.8‐60.0)	6	0	0.0 (0.0‐45.9)
*Tertiary ALI definition*	*9*	*2*	*22.2 (2.8*‐*60.0)*	*5*	*0*	*0.0 (0.0*‐*52.2)*
573.9 Unspecified disorders of liver						
Secondary ALI definition	1	0	0.0 (0.0‐97.5)	0	0	‐
*Tertiary ALI definition*	*0*	*0*	*‐*	*0*	*0*	*‐*
782.4 Jaundice, unspecified, not of newborn						
Secondary ALI definition	1	1	100 (2.5‐100)	2	2	100 (15.8‐100)
*Tertiary ALI definition*	*1*	*1*	*100 (2.5*‐*100)*	*2*	*2*	*100 (15.8*‐*100)*
V42.7 Liver transplant						
Secondary ALI definition	0	‐	‐	0	‐	‐
*Tertiary ALI definition*	*0*	*‐*	*‐*	*0*	*‐*	*‐*
790.4 Nonspecific elevation of transaminase or LDH						
Secondary ALI definition	1	1	100.0 (2.5‐100.0)	2	0	0.0 (0.0‐84.2)
*Tertiary ALI definition*	*1*	*1*	*100.0 (2.5*‐*100.0)*	*1*	*0*	*0.0 (0.0*‐*97.5)*
789.1 Hepatomegaly						
Secondary ALI definition	0	‐	‐	0	0	‐
*Tertiary ALI definition*	*0*	*‐*	*‐*	*0*	*0*	*‐*

Abbreviations: CI, confidence interval; ICD‐9‐CM, *International Classification of Diseases, Ninth Revision, Clinical Modification*; LDH, lactic acid dehydrogenase; TP, true positives.

Note: PPVs for the ICPC codes used to define cases for the tertiary ALI definition in EpiChron are presented in eTable 5.

a PPV was calculated as PPV = confirmed cases / (true positives + false positives). Results are presented as positive predictive values (%) and their 95% CIs.

**Table 4:** Positive predictive values (PPVs) of specific and nonspecific codes used to identify potential acute liver injury (ALI) cases: Secondary (regular font) and tertiary (italics) ALI definitions in data sources using ICD‐10‐CM codes (nonevaluable cases not included)

	SIDIAP^a^			Denmark^b^		
	Total	TP	PPV, % (95% CI)^c^	Total	TP	PPV, % (95% CI)^c^
Specific codes						
K71.0 Toxic liver disease with cholestasis						
Secondary ALI definition				n < 5	n < 5	50.0 (1.3–98.7)
*Tertiary ALI definition*	*0*	*‐*	*‐*	5	n < 5	*60.0 (14.7‐94.7)*
K71.1 Toxic liver disease with hepatic necrosis						
Secondary ALI definition				5	n < 5	40.0 (5.3*‐*85.3)
*Tertiary ALI definition*	*0*	*‐*	*‐*	6	n < 5	*33.3 (4.3‐77.7)*
K71.2 Toxic liver disease with acute hepatitis						
Secondary ALI definition				9	8	88.9 (51.8*‐*99.7)
*Tertiary ALI definition*	*0*	*‐*	*‐*	13	12	*92.3 (64.0‐99.8)*
K71.6 Toxic liver disease with hepatitis, not elsewhere classified						
Secondary ALI definition				8	7	87.5 (47.3*‐*99.7)
Tertiary ALI definition	5	2	40.0 (5.3*‐*85.3)	9	8	88.9 (51.8*‐*99.7)
K71.9 Toxic liver disease, unspecified						
Secondary ALI definition				5	n < 5	80.0 (28.4*‐*99.5)
*Tertiary ALI definition*	*1*	*0*	*0.0 (0.0‐97.5)*	*12*	6	*50.0 (21.1‐78.9)*
K72.0 Acute and subacute hepatic failure						
Secondary ALI definition				7	6	85.7 (42.1*‐*99.6)
*Tertiary ALI definition*	*3*	*2*	*66.7 (9.4‐99.2)*	*9*	8	*88.9 (51.8‐99.7)*
K72.9 Hepatic failure, unspecified						
Secondary ALI definition				10	6	60.0 (26.2*‐*87.8)
*Tertiary ALI definition*	*8*	*1*	*12.5 (0.3‐52.7)*	*13*	7	*53.8 (25.1‐80.8)*
K75.9 Inflammatory liver disease, unspecified						
Secondary ALI definition				n < 5	n < 5	66.7 (9.4*‐*99.2)
*Tertiary ALI definition*	*23*	*7*	*30.4 (13.2‐52.9)*	*5*	n < 5	*60.0 (14.7‐94.7)*
K76.2 Central hemorrhagic necrosis of liver						
Secondary ALI definition				n < 5	n < 5	100 (2.5*‐*100)
*Tertiary ALI definition*	*0*	*‐*	*‐*	n < 5	n < 5	*100 (2.5‐100)*
Nonspecific codes						
K76.8 Other specified diseases of liver						
Secondary ALI definition				16	n < 5	6.3 (0.2*‐*30.2)
*Tertiary ALI definition*	*111*	*1*	*0.9 (0.0‐4.9)*	35	n < 5	*11.4 (3.2‐26.7)*
K76.9 Liver disease, unspecified						
Secondary ALI definition				30	15	50.0 (31.3*‐*68.7)
*Tertiary ALI definition*	*116*	*11*	*9.5 (4.8‐16.3)*	*107*	33	*30.8 (22.3‐40.5)*
R17 Unspecified jaundice, excludes neonatal						
Secondary ALI definition				79	75	94.9 (87.5*‐*98.6)
*Tertiary ALI definition*	*57*	*20*	*35.1 (22.9‐48.9)*	*90*	82	*91.1 (83.2‐96.1)*
R16.0 Hepatomegaly, not elsewhere classified						
Secondary ALI definition				7	n < 5	42.9 (9.9*‐*81.6)
*Tertiary ALI definition*	*52*	*3*	*5.8 (1.2‐15.9)*	*12*	n < 5	*25.0 (5.5‐57.2)*
R16.2 Hepatomegaly with splenomegaly, not elsewhere classified						
Secondary ALI definition				n < 5	n < 5	75.0 (19.4*‐*99.4)
*Tertiary ALI definition*	*0*	*‐*	*‐*	*6*	n < 5	*50.0 (11.8‐88.2)*
R74.0 Nonspecific elevation of transaminase and LDH						
Secondary ALI definition				27	16	59.3 (38.8*‐*77.6)
*Tertiary ALI definition*	*1,852*	*119*	*6.4 (5.4‐7.6)*	*120*	33	*27.5 (19.7‐36.4)*
Z94.4 Liver transplant						
Secondary ALI definition				0	‐	‐
*Tertiary ALI definition*	*0*	*‐*	*‐*	*0*	‐	*‐*

Abbreviations: CI, confidence interval; ICD‐10‐CM, *International Classification of Diseases, Tenth Revision, Clinical Modification*; LDH, lactic acid dehydrogenase; TP, true positives.

a In SIDIAP, ICD‐10 codes were used only for the outpatient codes of the study tertiary ALI definition.

b Due to data protection policies in Denmark, the exact number of cases could not be provided when the number of cases was less than five.

c PPV was calculated as PPV = confirmed cases / (true positives + false positives). Results are presented as positive predictive values (%) and their 95% CIs.

For the tertiary ALI definition, the overall PPVs were 25% (95% CI, 18%‐34%) in EpiChron, 8% (95% CI, 7%‐9%) in SIDIAP, and 47% (95% CI, 42%‐52%) in Denmark. As observed for the secondary ALI definition, we observed higher PPVs for specific than nonspecific codes in all data sources (Table 2). Among the individual specific codes, 570.x (acute and subacute necrosis of liver) had the highest PPV in EpiChron and SIDIAP (Table 3 and Supplementary eTable S5). In Denmark, code K71.2 (toxic liver disease with acute hepatitis) had the highest PPV among specific codes (Table 4). Among the nonspecific codes, 782.4 (jaundice, unspecified, not of newborn) had the highest PPV in both EpiChron and SIDIAP, although it had a low number of confirmed cases (one and two true positives in EpiChron and SIDIAP, respectively). In Denmark, ICD‐10 code R17 (unspecified jaundice, excludes neonatal) had the highest PPV (91%) and contributed the largest number of true positives. In SIDIAP, the same code used to identify primary care diagnoses had the second highest PPV, and it was also the second highest contributor of true positives. Regarding code R74.0 (nonspecific elevation of transaminase or LDH), in SIDIAP, it was the code with the highest number of true positives, although it had a low PPV (6%).

In the sensitivity analysis including nonevaluable cases in the denominator of the PPV calculation, the overall PPVs for all study ALI definitions and for both specific and nonspecific codes were smaller than those for the main PPV analysis in all data sources (see Supplementary eTables S6 and S7).

## 4 DISCUSSION

We observed consistently higher overall PPVs for specific ALI codes versus nonspecific codes and higher overall PPVs for hospital discharge codes versus outpatient codes. The identification of ALI cases based on hospital discharge specific codes, considered as the primary ALI definition in this study, resulted in higher PPVs when compared with most previously described algorithms.^3^, ^4^, ^5^, ^6^

In contrast to the present study, previous studies conducted to validate ALI cases have reported PPVs below 60%,^3^, ^4^, ^5^ or around 75%.^6^ A recently published systematic review and meta‐analysis including 29 studies validating ALI or drug‐induced liver injury (DILI) (25 of them presenting PPVs) showed a pooled PPV estimate for ALI of 13.4% (95% CI, 6.1%‐22.8%) and for DILI of 15.3% (95% CI, 9.5%‐22.2%).^21^ The authors of that study suggested that the low PPVs observed in the studies might be explained by the low prevalence of ALI or DILI. In addition, a different list of diagnosis codes, laboratory threshold criteria, and study drugs might be the cause of the differences between studies. When we compared our study with previous studies validating ALI definitions, we observed that our study differed from these previous studies in different ways: Bui et al^6^ did not exclude patients with hepatic, biliary, or pancreatic diseases or cancer; Lo Re et al^3^ included only cases of severe ALI; Udo et al^5^ validated cases of idiopathic ALI only; and Traversa et al^4^ validated cases of ALI associated with the use of nonsteroidal antiinflammatory drugs. In addition, there are differences in the type of data sources: the Bui et al^6^ and Lo Re et al^3^ studies were conducted in claims databases including inpatient and outpatient encounters, prescriptions, and laboratory tests. The Traversa et al^4^ and Udo et al^5^ studies were conducted in hospital databases in a way similar to the Danish component of our study. There are also differences in the ALI definition used in previous studies compared with the criteria used in our study, which were based on Aithal criteria.^19^

Positive predictive values obtained in the present study for the ICD‐9 specific codes 573.3 (hepatitis unspecified) and 570.x (acute and subacute necrosis of liver) and specific ICD‐10 codes K71.2 (toxic liver disease with acute hepatitis) and K71.6 (toxic liver disease with hepatitis, not elsewhere specified) were in line with previous studies. In Udo et al,^5^ the code 573.3 had a PPV of 80%. In Bui et al,^6^ the PPV for individual code 570.x was 84% and for 573.3 was 76%, while the PPV for the algorithm including codes 570.x, 572.2 (hepatic coma), or 573.3 was 74%. In Lo Re et al,^3^ the PPVs for individual codes ranged from 6.5% to 54.3%, the combination of codes 570.x with 572.8 (sequelae of liver disease; hepatic failure) had a PPV of 100%, and code 570.x in combination with 572.2 had a PPV of 67%. In addition, the authors calculated PPVs including patients with preexisting liver disease, and the PPVs were higher when compared with the subset of the population that excluded those patients.^3^ In two studies validating drug‐induced ALI (DILI),^22^, ^23^ code 573.3 (hepatitis unspecified) was the highest contributor of DILI cases.

In the present study, the nonspecific code for unspecified jaundice (R17) obtained high PPVs, and it was the highest contributor of true positives in Denmark. In EpiChron and SIDIAP databases, the ICD‐9‐CM code 782.4 (jaundice, unspecified, not of newborn) had high PPVs for the secondary ALI definition (hospitalized cases), although the number of true positives was one and two cases, respectively. In SIDIAP, the ICD‐10 code for unspecified jaundice used in the tertiary ALI definition to validate hospitalized and outpatient cases was the second contributor of true positives and had the second‐highest PPV, although it was low (35%). Potential explanations for this discrepancy in the results for unspecified jaundice code between Denmark and Spanish data sources could be the following: (a) in Denmark, only hospitalized and outpatient cases from hospital outpatient clinics are validated; and (b) in Denmark, exclusion criteria not identified previously were applied, if identified, during either the abstraction or the review of the abstracted information from medical records. These reasons may reduce the presence of false positives and justify the high PPV observed for this code in Denmark compared with Spanish data sources. Results observed in Denmark also contrast with those in a previous study,^23^ which reported that the nonspecified code for unspecified jaundice identified only a small proportion of DILI cases (5% of the 265 cases in Shin et al^23^ vs 39% of the 208 cases of the tertiary ALI definition confirmed in Denmark observed in our study), but the differences when validating ALI or DILI cases must be taken into account. In addition, the study by Shin et al^23^ was not restricted to hospital cases as it was in Denmark, and thus the prevalence of true ALI in populations including outpatient primary care cases should be lower, which would explain the differences observed between the two studies.

### 4.1 Strengths and limitations

In terms of number of validated cases, the present validation study represents one of the largest efforts performed in Europe to validate ALI cases identified in automated health care databases, using case‐identifying algorithms, and confirmed according to consensus criteria based on the presence of elevated liver enzyme levels in blood. In addition, to the best of our knowledge, this study is the first to validate ICD‐10 codes related to ALI. However, the results obtained in the present study must be evaluated in the context of its limitations. An important limitation of this study is that, although the ALI definitions were consistent across data sources and based on blood liver enzyme levels, the approach to the evaluation of potential cases was adapted to the type of information and local resources available for the validation efforts, which may have impacted our findings. In SIDIAP, the validation was partial for all potential cases (inpatient and outpatient), based only on liver enzyme results from primary care, and no hospital medical records to validate hospital cases were available. That could explain the lowest PPV for the secondary ALI definition in SIDIAP. In Denmark, only outpatient potential cases from hospital outpatient clinics could be identified (primary care data were not available). This is probably the reason why the difference in PPVs between specific and nonspecific codes was smaller in Denmark than in the other data sources, and it would also explain the higher PPVs obtained in Denmark for the secondary and tertiary ALI definitions compared with the two Spanish data sources. For some codes, the number of cases was low, resulting in wide CIs for the PPV. The present study has also other limitations. First, we did not conduct validation of false positives, and therefore negative predictive values could not be estimated. Second, the PPVs obtained in the present study apply only to patients using the study antidepressant drugs who did not have preexisting liver disease or risk factors for developing ALI. Third, PPVs are dependent on the ALI case definition used. In the present study, we used the definition created by Aithal et al,^19^ but there are other case definitions that could be used^24^, ^25^ and PPVs could have been different with those other case definition criteria. Finally, PPVs are dependent on ALI prevalence. Therefore, the PPVs observed in our study might not apply directly to patient populations with characteristics different from those included in the present study or to studies using different case definitions.

## 5 CONCLUSIONS

The PPVs obtained in this study apply to patients using antidepressants without preexisting liver disease or risk factors for ALI. Future studies evaluating ALI in these and similar data sources should prioritize use of hospital discharge and specific codes to maximize validity. Moreover, case‐identifying algorithms should include hospital ICD codes for unspecified jaundice. In studies including nonspecific codes and outpatient cases, case validation is essential.

## ETHICS STATEMENT

RTI International institutional review board approval to conduct the study was granted on 04 August 2015. The following data source‐specific approvals were obtained: in EpiChron, Ethics committee approval was obtained from the Comité Etico de Investigación Clínica de Aragón (07 October 2015) and the Spanish Agency of Medicines and Medical Devices (AEMPS) (08 September 2015). In SIDIAP, ethics committee approval was obtained from the IDIAP Jordi Gol Ethics Committee (23 December 2015). In Denmark, the authorization of the Danish Data Protection Agency was granted on 15 June 2015. The Danish Health Authority provided authorization to access medical records, granted on 29 March 2016.

## CONFLICT OF INTEREST

Manel Pladevall, Joan Forns, Miguel Cainzos‐Achirica, Jordi Castellsagué, and Susana Perez‐Gutthann are employees of RTI Health, a unit of RTI International, a nonprofit organization that conducts work for government, public, and private organizations, including pharmaceutical companies.

Alexandra Prados‐Torres and Beatriz Poblador‐Plou are members of the EpiChron Research Group on Chronic Diseases of the Aragon Health Sciences Institute (IACS), ascribed to IIS Aragón, and do not have any conflict of interest with this project.

Maria Giner‐Soriano, Rosa Morros, and Jordi Cortés worked on other projects funded by pharmaceutical companies in their institution that were not related to this study and without personal profit.

Anton Pottegård reports participation in research projects funded by Alcon, Almirall, Astellas, AstraZeneca, and Servier, all with funds paid to the institution where he was employed (no personal fees) and with no relation to the work reported in this paper.

Jesper Hallas has participated in research projects funded by Novartis, Pfizer, Menarini, MSD, Nycomed, LEO Pharma, Almirall, Servier, Astellas, and Alkabello with grants paid to the institution where he was employed. He has personally received fees for teaching or consulting from the Danish Association of Pharmaceutical Manufacturers and from Pfizer and Menarini.

Maja Hellfritzsch has received speaker honorarium fees from Bristol‐Myers Squibb and Pfizer and a travel grant from LEO Pharma.

Nicolas Deltour and Emmanuelle Jacquot are employees of Les Laboratoires Servier.

## FUNDING

This study was funded by Les Laboratoires Servier under a contract granting independent publication rights to the research team.

## DATA AVAILABILITY

The data sets used for this study are owned by each of the individual research center or by the government data custodians from which the research centers obtained access to the data at IACS (Spain), SIDIAP (SIDIAP), BIPS (Germany), Karolinska Institutet (Sweden), and Southern Denmark University (Denmark). Researchers desiring access to the data sets would be required to obtain permission from research center and/or data custodians at each country. Researchers desiring access to the code used to analyze that data would be required to obtain permission from the research centers and the study sponsor.

## AUTHORS' CONTRIBUTIONS

Authors Manel Pladevall, Jordi Castellsagué, Emmanuelle Jacquot, Nicolas Deltour, and Susana Perez‐Gutthann planned the study. All authors made contributions to the final design and final approved version of the protocol. Authors Alexandra Prados‐Torres, Beatriz Poblador‐Plou, Maria Giner‐Soriano, Rosa Morros, Jordi Cortés, Anton Pottegård, Jesper Hallas, and Maja Hellfritzsch undertook the statistical analysis of the different data sources. Joan Forns, Miguel Cainzos‐Achirica, Susana Perez‐Gutthann, and Manel Pladevall wrote the first draft of the manuscript. All authors contributed to and have approved the final manuscript.

## Supporting information

Table S1. Characteristics of the data sources
Table S2. Information available in each data source at step 1 and step 2 and implications of overall data availability for validation of secondary and tertiary ALI definitions
Table S3. Summary of the cohort attrition for users of study antidepressants in the agomelatine PASS in the three databases with validation activities
Table S4. Age and sex distribution of the true positives for the tertiary ALI definition
Table S5. Positive predictive values (PPVs) of nonspecific codes used to identify potential cases of acute liver injury: tertiary ALI definition in data sources using ICPC codes (nonevaluable cases not included)
Table S6.Positive predictive values (PPVs) of specific and nonspecific codes used to identify potential acute liver injury (ALI) cases: secondary ALI definition, sensitivity analysis (nonevaluable cases included)
Table S7.Positive predictive values (PPVs) of specific and nonspecific codes used to identify potential acute liver injury (ALI) cases: tertiary ALI definition, sensitivity analysis (nonevaluable cases included)

## References

[1] Hussaini SH , Farrington EA . Idiosyncratic drug‐induced liver injury: an update on the 2007 overview. Expert Opin Drug Saf. 2014;13(1):67‐81. 10.1517/14740338.2013.828032 24073714

[2] Lee WM , Stravitz RT , Larson AM . Introduction to the revised American Association for the Study of Liver Diseases Position Paper on acute liver failure 2011. Hepatology. 2012;55(3):965‐967. 10.1002/hep.25551 22213561 PMC3378702

[3] Lo Re V 3rd , Haynes K , Goldberg D , et al. Validity of diagnostic codes to identify cases of severe acute liver injury in the US Food and Drug Administration's mini‐sentinel distributed database. Pharmacoepidemiol Drug Saf. 2013;22(8):861‐872. 10.1002/pds.3470 23801638 PMC4409951

[4] Traversa G , Bianchi C , Da Cas R , Abraha I , Menniti‐Ippolito F , Venegoni M . Cohort study of hepatotoxicity associated with nimesulide and other non‐steroidal anti‐inflammatory drugs. BMJ. 2003;327(7405):18‐22. 10.1136/bmj.327.7405.18 12842950 PMC164233

[5] Udo R , Maitland‐van der Zee AH , Egberts TC , et al. Validity of diagnostic codes and laboratory measurements to identify patients with idiopathic acute liver injury in a hospital database. Pharmacoepidemiol Drug Saf. 2016;25(Suppl 1):21‐28. 10.1002/pds.3824 26147715

[6] Bui CL , Kaye JA , Castellsague J , et al. Validation of acute liver injury cases in a population‐based cohort study of oral antimicrobial users. Curr Drug Saf. 2014;9(1):23‐28. 24111729 10.2174/15748863113086660051

[7] Pladevall‐Vila M. Post‐authorisation safety study of agomelatine and the risk of hospitalisation for acute liver injury 16/01/2018 2018 http://www.encepp.eu/encepp/viewResource.htm?id=12730 (accessed 2 March 2018).

[8] Prados‐Torres A , Poblador‐Plou B , Gimeno‐Miguel A , et al. Cohort profile: the epidemiology of chronic diseases and multimorbidity. The EpiChron cohort study. Int J Epidemiol. 2018;47(2):382‐384f. 10.1093/ije/dyx259 29346556 PMC5913592

[9] SIDIAP. Database . General details. 2014 http://www.sidiap.org/index.php/en (accessed 27 September 2016).

[10] Schmidt M , Schmidt SA , Sandegaard JL , Ehrenstein V , Pedersen L , Sorensen HT . The Danish National Patient Registry: a review of content, data quality, and research potential. Clin Epidemiol. 2015;7:449‐490. 10.2147/clep.s91125 26604824 PMC4655913

[11] Pottegard A , Schmidt SA , Wallach‐Kildemoes H , Sorensen HT , Hallas J , Schmidt M . Data resource profile: the Danish National Prescription Registry. Int J Epidemiol. 2016 10.1093/ije/dyw213 PMC5837522 27789670

[12] Pigeot I , Ahrens W . Establishment of a pharmacoepidemiological database in Germany: methodological potential, scientific value and practical limitations. Pharmacoepidemiol Drug Saf. 2008;17(3):215‐223. 10.1002/pds.1545 18200610

[13] Jobski K , Kollhorst B , Garbe E , Schink T . The risk of ischemic cardio‐ and cerebrovascular events associated with oxycodone‐naloxone and other extended‐release high‐potency opioids: a nested case‐control study. Drug Saf. 2017;40(6):505‐515. 10.1007/s40264-017-0511-8 28194654

[14] Jobski K , Schmedt N , Kollhorst B , Krappweis J , Schink T , Garbe E . Characteristics and drug use patterns of older antidepressant initiators in Germany. Eur J Clin Pharmacol. 2017;73(1):105‐113. 10.1007/s00228-016-2145-7 27752752

[15] Ludvigsson JF , Andersson E , Ekbom A , et al. External review and validation of the Swedish national inpatient register. BMC Public Health. 2011;11(1):450 10.1186/1471-2458-11-450 21658213 PMC3142234

[16] Wettermark B , Hammar N , Fored CM , et al. The new Swedish prescribed Drug register—opportunities for pharmacoepidemiological research and experience from the first six months. Pharmacoepidemiol Drug Saf. 2007;16(7):726‐735. 10.1002/pds.1294 16897791

[17] Timmer A , Kappen S , de Sordi D , Pladevall M , Perez‐Gutthann S , Jacquot E , Deltour N , Schink T . Validity of hospital ICD‐10‐GM codes to identify acute liver injury. Presented at the 34th International Conference on Pharmacoepidemiology and Therapeutic Risk Management; August 22–26 2018. Prague, Czech Republic.p. 262.

[18] Pladevall‐Vila M , Pottegard A , Schink T , et al. Risk of acute liver injury in agomelatine and other antidepressant users in four European countries: a cohort and nested case‐control study using automated health data sources. CNS Drugs. 2019;33(4):383‐395. 10.1007/s40263-019-00611-9 30830574 PMC6441103

[19] Aithal GP , Watkins PB , Andrade RJ , et al. Case definition and phenotype standardization in drug‐induced liver injury. Clin Pharmacol Ther. 2011;89(6):806‐815. 10.1038/clpt.2011.58 21544079

[20] StataCorp LP . Stata User's Guide: Release 14. College Station, Tex: StataCorp LP; 2015.

[21] Tan EH , Low EXS , Dan YY , Tai BC . Systematic review and meta‐analysis of algorithms used to identify drug‐induced liver injury (DILI) in health record databases. Liver Int. 2018;38(4):742‐753. 10.1111/liv.13646 29193566

[22] Jinjuvadia K , Kwan W , Fontana RJ . Searching for a needle in a haystack: use of ICD‐9‐CM codes in drug‐induced liver injury. Am J Gastroenterol. 2007;102(11):2437‐2443. 10.1111/j.1572-0241.2007.01456.x 17662100

[23] Shin J , Hunt CM , Suzuki A , Papay JI , Beach KJ , Cheetham TC . Characterizing phenotypes and outcomes of drug‐associated liver injury using electronic medical record data. Pharmacoepidemiol Drug Saf. 2013;22(2):190‐198. 10.1002/pds.3388 23258383

[24] Bénichou C . Criteria of drug‐induced liver disordersReport of an international consensus meeting. J Hepatol. 1990;11(2):272‐276. 2254635 10.1016/0168-8278(90)90124-a

[25] FDA . Drug‐Induced Liver Injury: Premarketing Clinical Evaluation. US Food and Drug Administration, 2009 https://www.fda.gov/downloads/guidances/UCM174090.pdf (accessed 1 June 2018).

