# Supplementary material for: Validity of ICD‐9 and ICD‐10 codes used to identify acute liver injury: A study in three European data sources
Source: Pharmacoepidemiol Drug Saf. 2019 Jun 6;28(7):965–75. doi: 10.1002/pds.4803 (PMC6618105; doi:10.1002/pds.4803)
Supplement: Supplementary file 1 — Table S1. Characteristics of the data sources Table S2. Information available in each data source at step 1 and step 2 and implications of overall data availability for validation of secondary and tertiary ALI definitions Table S3. Summary of the cohort attrition for users of study antidepressants in the agomelatine PASS in the three databases with validation activities Table S4. Age and sex distribution of the true positives for the tertiary ALI definition Table S5. Positive predictive values (PPVs) of nonspecific codes used to identify potential cases of acute liver injury: tertiary ALI definition in data sources using ICPC codes (nonevaluable cases not included) Table S6.Positive predictive values (PPVs) of specific and nonspecific codes used to identify potential acute liver injury (ALI) cases: secondary ALI definition, sensitivity analysis (nonevaluable cases included) Table S7.Positive predictive values (PPVs) of specific and nonspecific codes used to identify potential acute liver injury (ALI) cases: tertiary ALI definition, sensitivity analysis (nonevaluable cases included) [file PDS-28-965-s001.docx]

Supplementary eTable 1. Characteristics of the Data Sources

| Database Feature | EpiChron (Spain) | SIDIAP (Spain) | Danish National Health Registers (Denmark) |
| --- | --- | --- | --- |
| Database population (% of population covered in the country) | 1,344,891 (3%) | 2,066,576 (4%) | 5,627,235 (100%) |
| Database type | Primary health care electronic medical record database; link to hospital discharge data and pharmacy data | Primary health care electronic medical record database; link to hospital discharge data, pharmacy data, and mortality data | National health record databases; link to other national databases through the unique Civil Personal Registration Number |
| Disease and procedure coding system(s) | Primary health care, ICPC; hospital, ICD‑9‑CM | ICD‑10‑CM for primary care diagnoses; ICD‑9‑CM for hospital diagnoses | ICD‑10 |
| Data available for validation | |  |  |
| Primary care | Yes | Yes | No |
| Specialist outpatient visits | Yes | Only referrals from GPs to hospital specialists are recorded (for the first visit to specialists, only scheduled date is recorded) | Only hospital clinic visits |
| Hospital discharge | Yes | Yes | Yes |
| Laboratory (liver enzyme results) | Yes (primary care and hospital via abstraction of medical records) | Only primary care results | Results available only via abstraction of medical records |
| Access to hospital medical records | Yes | No | Yes |

GP = general practitioner; ICD‑9‑CM = *International Classification of Diseases, Ninth Revision, Clinical Modification*; ICD‑10‑CM = *International Classification of Diseases, Tenth Revision, Clinical Modification*; ICPC = International Classification of Primary Care.

Supplementary eMethods. Study Exclusion Criteria

To control for potential confounding factors, the study cohort was restricted to patients without a history of liver disease or risk factors for liver disease. Therefore, patients with any of the listed conditions recorded as primary or secondary discharge diagnoses at any time before the start date were excluded from the study:

- Acute and subacute liver disease, including viral and other infectious or toxic hepatitis
- Chronic liver diseases, such as cirrhosis or fibrosis of the liver, alcoholic liver disease, chronic toxic liver disease, hemochromatosis, Wilson disease, deficit of alpha-1-antitrypsin, and Budd-Chiari syndrome
- Disorders of bilirubin excretion such as Gilbert’s syndrome and Crigler-Najjar syndrome
- Chronic biliary or pancreatic disease
- Risk factors for liver disease: alcohol use disorder, heart failure
- Malignancy
- Human immunodeficiency virus (HIV) infection/AIDS
- Liver or other organ transplant
- Drug abuse and dependence
- History of paracetamol intoxication
- Jaundice (excluding neonatal jaundice)
- Hepatomegaly
- Other and unspecified disorders of the liver
- Nonspecific elevation of levels of transaminase and lactic acid dehydrogenase (LDH)

Patients with a history of infectious liver injury or HIV/AIDS (who have a higher risk of viral hepatitis than the general population) were excluded from the study cohort because the focus of this study is noninfectious acute liver injury.

Supplementary eTable 2. Information Available in Each Data Source at Step 1 and Step 2 and Implications of Overall Data Availability for Validation of Secondary and Tertiary ALI definitions

| Data Source | Review of Patient Profiles | | Abstraction of Data From Medical Records |
| --- | --- | --- | --- |
|  | Information Available | Information Used to… | Information Available and Implications for ALI definition Validation^a^ |
| EpiChron, Spain | - Hospital discharge ICD‑9 codes for all inpatient episodes during the study period - Prescription medications 12 months before and after the start date, up to end of follow-up - ICPC codes - Liver enzyme results from primary care visits^b^ | - Identify potential exclusion criteria missed by the case-identification algorithms - Classify as nonevaluable those potential cases without an ALI hospitalization and with no liver enzymes available from either primary care or hospital outpatient data sources (no additional data could be retrieved for validation) - Initially^c^ classify as noncases those potential cases without an ALI-related hospitalization whose liver enzymes did not meet validation criteria and those potential cases that met validation criteria but had biological evidence of chronic liver injury - Initially^c^ classify as probable cases those potential cases whose liver enzymes met validation criteria | All data from patient profile review plus:   - All abstracted data from the electronic health record (EHR). The EHR allows access to both primary care and hospital records, including the discharge summary and imaging reports. Data were abstracted from EHRs using an electronic data capture (EDC) system developed with Microsoft Access software - Liver enzyme results from inpatient and outpatient hospital episodes were abstracted from the EHR   **Implications of overall data availability for ALI definition validation:**   - Validation of both secondary and tertiary ALI definitions was feasible (results for all liver tests performed, regardless of the setting, were available) |
| SIDIAP, Spain | - Hospital discharge ICD‑9‑CM ALI definition codes - ICD‑10‑CM primary care codes - Liver enzyme results available in primary care records | - Classify as nonevaluable those potential cases with no liver enzymes available - Initially classify as noncases those potential cases whose liver enzymes did not meet validation criteria - Initially classify as possible or probable cases those potential cases whose liver enzymes met validation criteria | Hospital medical record abstraction was not feasible; therefore, in-hospital liver enzyme results were not available   - All data reviewed were from patient profiles   **Implications of overall data availability for ALI definition validation:**   - Partial validation of secondary ALI definition: liver enzyme results from primary care data sources were available for approximately 50% of hospitalized potential cases - Partial validation of tertiary ALI definition: for outpatient hospital visits, liver enzyme results were not available; for non-hospitalized patients, liver enzyme results were available for approximately 70%% of potential cases |
| DNHR, Denmark | - Hospital discharge ICD‑10 codes for all inpatient episodes during the study period - Hospital outpatient ICD‑10 codes for all outpatient episodes during the study period - Prescription data on drugs used to treat alcoholism | - Patient profile review was not implemented in the DNHR because of the limited availability of data essential for case classification | - Hospital records for both outpatient and inpatient episodes. Records of all potential cases identified by the electronic algorithms for which medical records could be retrieved were reviewed, and relevant data were abstracted using an EDC system (REDCap; <http://projectredcap.org/>). When an exclusion criterion not previously known was identified during medical record abstraction, the process of abstraction was stopped and the potential case was excluded from the study - Secondary care and primary care liver enzyme results were abstracted from hospital medical records when available   **Implications of overall data availability for ALI definition validation:**   - Validation of the secondary ALI definition was feasible - Validation of the tertiary ALI definition: primary care codes were not available |

ALI = acute liver injury; DNHR = Danish National Health Registers; EDC = electronic data capture; EHR = electronic health record; ICD‑9 = *International Classification of Diseases, Ninth Revision*; ICD‑9‑CM = *International Classification of Diseases, Ninth Revision, Clinical Modification;* ICD‑10 = *International Classification of Diseases, Tenth Revision*; ICD‑10‑CM = *International Classification of Diseases, Tenth Revision, Clinical Modification*; ICPC = International Classification of Primary Care.

^a^ All information available after the medical record review was used for final case classification.

^b^ EpiChron in Spain: outpatient laboratory values are available since 2010.

^c^ Here, “initially” refers to the fact that the initial classification was reviewed after medical record abstraction.

Supplementary eTable 3. Summary of the Cohort Attrition for Users of Study Antidepressants in the Agomelatine PASS in the three databases with Validation Activities

|  | EpiChron | | SIDIAP | | Denmark | |
| --- | --- | --- | --- | --- | --- | --- |
|  | All  Users^a^ | New Users  Included^b^ | All  Users^a^ | New Users  Included^b^ | All  Users^a^ | New Users Included^b^ |
| Citalopram | 19,299 | 9,016 (46.7%) | 69,533 | 41,295 (59.4%) | 356,097 | 199,887 (56.1%) |
| Agomelatine | 10,700 | 8,826 (82.5%) | 4,686 | 3,243 (69.2%) | 22,131 | 18,032 (81.5%) |
| Fluoxetine | 27,406 | 15,379 (56.1%) | 36,306 | 19,235 (53.0%) | 31,601 | 15,475 (49.0%) |
| Paroxetine | 43,800 | 23,570 (53.8%) | 58,326 | 31,392 (53.8%) | 32,311 | 15,581 (48.2%) |
| Sertraline | 22,177 | 11,054 (49.8%) | 37,382 | 21,148 (56.6%) | 167,973 | 113,861 (67.8%) |
| Escitalopram | 74,591 | 46,129 (61.8%) | 42,911 | 23,107 (53.8%) | 99,190 | 44,817 (45.2%) |
| Mirtazapine | 34,876 | 19,402 (55.6%) | 25,874 | 14,161 (54.7%) | 205,378 | 124,647 (60.7%) |
| Venlafaxine | 22,748 | 10,577 (46.5%) | 24,960 | 11,840 (47.4%) | 116,798 | 71,649 (61.3%) |
| Duloxetine | 32,314 | 20,899 (64.7%) | 19,281 | 10,368 (53.8%) | 49,504 | 30,212 (61.0%) |
| Amitriptyline | 31,797 | 20,776 (65.3%) | 42,562 | 27,312 (64.2%) | 46,261 | 30,044 (64.9%) |
| **Total** | **319,708** | **185,628 (58.1%)** | **361,821** | **203,101 (56.1%)** | **1,127,244** | **664,205 (58.9%)** |

PASS = post-authorisation safety study.

^a^ Total number of users before applying eligibility and exclusion criteria.

^b^ Total number of users after applying eligibility and exclusion criteria. Percentages in this column are row percentages of the total number of all users.

Supplementary eTable 4. Age and Sex Distribution of the True Positives for the Tertiary ALI definition

|  | EpiChron  (N = 34) | SIDIAP  (N = 172) | Denmark  (N = 208) |
| --- | --- | --- | --- |
| Sex |  |  |  |
| Male | 15 (44.1%) | 39 (22.7%) | 61 (29.3%) |
| Female | 19 (55.9%) | 133 (77.3%) | 147 (70.7%) |
| Age, years |  |  |  |
| 18-29 | 2 (5.9%) | 6 (3.5%) | 9 (4.3%) |
| 30-39 | 3 (8.8%) | 12 (7.0%) | 18 (8.7%) |
| 40-49 | 4 (11.8%) | 30 (17.4%) | 23 (11.1%) |
| 50-59 | 6 (17.7%) | 33 (19.2%) | 31 (14.9%) |
| 60-69 | 6 (17.7%) | 31 (18.0%) | 27 (13.0%) |
| 70-79 | 5 (14.7%) | 26 (15.1%) | 43 (20.7%) |
| ≥ 80 | 8 (23.5%) | 34 (19.8%) | 57 (27.4%) |

Supplementary eTable 5. Positive Predictive Values (PPVs) of Nonspecific Codes Used to Identify Potential Cases of Acute Liver Injury: Tertiary ALI definition in Data Sources Using ICPC Codes (Nonevaluable Cases Not Included)

|  | EpiChron | | |
| --- | --- | --- | --- |
|  | Total | TP. | PPV, % (95% CI) |
| **Code** | | | |
| D97 Liver disease (specified or unspecified) | 44 | 8 | 18.2 (8.2-32.7) |
| D13 Jaundice | 35 | 7 | 20.0 (8.4-36.9) |
| D23 Hepatomegaly | 24 | 0 | 0.0 (0.0-14.3) |
| A91 Abnormal results investigations | 2 | 0 | 0.0 (0.0-84.2) |

CI = confidence interval; ICPC = International Classification of Primary Care; TP = True positives.

Note: PPV was calculated as PPV = true positives cases / (true positives+ false positives). Results are presented as positive predictive values (%) and their 95% CIs.

Supplementary eTable 6. Positive Predictive Values (PPVs) of Specific and Nonspecific Codes Used to Identify Potential Acute Liver Injury (ALI) Cases: Secondary ALI definition, Sensitivity Analysis (Nonevaluable Cases Included)

|  | EpiChron PPV, % (95% CI) | SIDIAP  PPV, % (95% CI) | Denmark  PPV, % (95% CI) |
| --- | --- | --- | --- |
| **Any ALI code** | 50 (33.8‑66.2) | 23.5 (10.7‑41.2) | 62.2 (55.8‑68.4) |
| **Specific codes** | 80 (56.34‑94.3) | 31.6 (12.6‑56.6) | 62.7 (49.1‑75.0) |
| ICD-9-CM codes |  |  |  |
| 570.x Acute and subacute necrosis of liver | 100.0 (47.82‑100) | 30.0 (6.7‑65.2) | NA |
| 572.2 Hepatic coma | 0 (0‑84.19) | NA | NA |
| 573.3 Hepatitis unspecified | 84.62 (54.6‑98.1) | 33.3 (7.5‑70.1) | NA |
| ICD-10 codes |  |  |  |
| K71.0 Toxic liver disease with cholestasis | NA | NA | 33.3 (0.8‑90.6) |
| K71.1 Toxic liver disease with hepatic necrosis | NA | NA | 33.3 (4.3‑77.7) |
| K71.2 Toxic liver disease with acute hepatitis | NA | NA | 80.0 (44.4‑97.5) |
| K71.6 Toxic liver disease with hepatitis, not elsewhere classified | NA | NA | 87.5 (47.3‑99.7) |
| K71.9 Toxic liver disease, unspecified | NA | NA | 57.1 (18.4‑90.1) |
| K72.0 Acute and subacute hepatic failure | NA | NA | 66.7 (29.9‑92.5) |
| K72.9 Hepatic failure, unspecified | NA | NA | 50.0 (21.1‑78.9) |
| K75.9 Inflammatory liver disease, unspecified | NA | NA | 66.7 (9.4‑99.2) |
| K76.2 Central hemorrhagic necrosis of liver | NA | NA | 100.0 (2.5‑100.0) |
| **Nonspecific codes** | 20.0 (5.7‑43.7) | 13.3 (1.7‑40.5) | 62.1 (54.6‑69.2) |
| ICD-9-CM codes |  |  |  |
| 573.8 Other specified disorders of liver | 14.29 (1.8‑42.8) | 0.0 (0.0‑30.8) | NA |
| 573.9 Unspecified disorders of liver | 0 (0‑97.5) | NA | NA |
| 782.4 Jaundice, unspecified, not of newborn | 50 (1.3‑98.7) | 100.0 (15.8‑100.0) | NA |
| V42.7 Liver transplant | NA | NA | NA |
| 790.4 Nonspecific elevation of transaminase or lactic acid dehydrogenase | 33.33 (0.8‑90.6) | 0.0 (0.0‑70.8) | NA |
| 789.1 Hepatomegaly | NA | NA | NA |
| ICD-10 codes | NA | NA |  |
| K76.8 Other specified diseases of liver | NA | NA | 5.3 (0.1‑26.0) |
| K76.9 Liver disease, unspecified | NA | NA | 41.7 (25.5‑59.2) |
| R17 Unspecified jaundice, excludes neonatal | NA | NA | 86.2 (77.1‑92.7) |
| R16.0 Hepatomegaly, not elsewhere classified | NA | NA | 42.9 (9.9‑81.6) |
| R16.2 Hepatomegaly with splenomegaly, not elsewhere classified | NA | NA | 75.0 (19.4‑99.4) |
| R74.0 Nonspecific elevation of transaminase and lactic acid dehydrogenase | NA | NA | 55.2 (35.7‑73.6) |
| Z94.4 Liver transplant | NA | NA | NA |

CI = confidence interval; ICD-9-CM = *International Classification of Diseases, Ninth Revision, Clinical Modification*; ICD‑10 = *International Classification of Diseases, Tenth Revision*; ICPC = International Classification of Primary Care; NA = not applicable.

Note: Positive predictive value was calculated as PPV = true positives / (true positives + false positives + nonevaluable cases). Results are presented as PPVs (%) and their 95% CIs.

Supplementary eTable 7. Positive Predictive Values (PPVs) of Specific and Nonspecific Codes Used to Identify Potential Acute Liver Injury (ALI) Cases: Tertiary ALI definition, Sensitivity Analysis (Nonevaluable Cases Included)

|  | EpiChron  PPV, % (95% CI) | SIDIAP  PPV, % (95% CI) | Denmark  PPV, % (95% CI) |
| --- | --- | --- | --- |
| **Any ALI code** | 14.3 (10.1‑19.4) | 6.1 (5.2‑7.0) | 40.9 (36.6‑45.3) |
| **Specific codes** | 79.0 (54.4‑94.0) | 19.5 (11.6‑29.7) | 59.5 (48.3‑70.1) |
| ICD-9-CM codes |  |  |  |
| 570.x Acute and subacute necrosis of liver | 100 (47.8‑100) | 16.7 (0.4‑64.1) | NA |
| 572.2 Hepatic coma | 0 (0‑84.2) | – | NA |
| 573.3 Hepatitis unspecified | 83.3 (51.59‑97.91) | 50.0 (11.8‑88.2) | NA |
| ICD-10 codes |  |  |  |
| K71.0 Toxic liver disease with cholestasis | NA | 0.0 (0.0‑97.5) | 50.0 (11.8‑88.2) |
| K71.1 Toxic liver disease with hepatic necrosis | NA | NA | 28.6 (3.7‑71.0) |
| K71.2 Toxic liver disease with acute hepatitis | NA | NA | 85.7 (57.2‑98.2) |
| K71.6 Toxic liver disease with hepatitis, not elsewhere classified | NA | 28.6 (3.7‑71.0) | 88.9 (51.8‑99.7) |
| K71.9 Toxic liver disease, unspecified | NA | 0.0 (0.0‑84.2) | 40.0 (16.3‑67.7) |
| K72.0 Acute and subacute hepatic failure | NA | 40.0 (5.3‑85.3) | 72.7 (39.0‑94.0) |
| K72.9 Hepatic failure, unspecified | NA | 5.6 (0.1‑27.3) | 46.7 (21.3‑73.4) |
| K75.9 Inflammatory liver disease, unspecified | NA | 20.0 (8.4‑36.9) | 50.0 (11.8‑88.2) |
| K76.2 Central hemorrhagic necrosis of liver | NA | NA | 100.0 (2.5‑100.0) |
| **Nonspecific codes** | 8.7 (5.3‑13.2) | 5.7 (4.8‑6.6) | 37.2 (32.6‑42.0) |
| ICD-9-CM codes |  |  |  |
| 573.8 Other specified disorders of liver | 14.3 (1.8‑42.8) | 0.0 (0.0‑33.6) | NA |
| 573.9 Unspecified disorders of liver | NA | NA | NA |
| 782.4 Jaundice, unspecified, not of newborn | 50 (1.3‑98.7) | 100.0 (15.8‑100.0) | NA |
| V42.7 Liver transplant | – | 0.0 (0.0‑97.5) | NA |
| 790.4 Nonspecific elevation of transaminase or lactic acid dehydrogenase | 33.3 (0.8‑90.6) | 0.0 (0.0‑84.2) | NA |
| 789.1 Hepatomegaly | NA | NA | NA |
| ICD-1 codes |  |  |  |
| K76.8 Other specified diseases of liver | NA | 0.4 (0.0‑2.1) | 9.5 (2.7‑22.6) |
| K76.9 Liver disease, unspecified | NA | 6.9 (3.5‑12.0) | 25.6 (18.3‑34.0) |
| R17 Unspecified jaundice, excludes neonatal | NA | 26.3 (16.9‑37.7) | 82.0 (73.1‑89.0) |
| R16.0 Hepatomegaly, not elsewhere classified | NA | 3.4 (0.7‑9.6) | 21.4 (4.7‑50.8) |
| R16.2 Hepatomegaly with splenomegaly, not elsewhere classified | NA | NA | 50.0 (11.8‑88.2) |
| R74.0 Nonspecific elevation of transaminase and lactic acid dehydrogenase | NA | 5.5 (4.6‑6.6) | 24.6 (17.6‑32.8) |
| Z94.4 Liver transplant | NA | 0.0 (0.0‑97.5) | NA |
| ICPC Codes |  |  |  |
| D97 Liver disease (specified or unspecified) | 11.0 (4.9‑20.5) | NA | NA |
| D13 Jaundice | 9.7 (4‑19.0) | NA | NA |
| D23 Hepatomegaly | 0 (0‑7.0) | NA | NA |
| A91 Abnormal results investigations | 0 (0‑60.2) | NA | NA |

CI = confidence interval; ICD-9-CM = *International Classification of Diseases, Ninth Revision, Clinical Modification*; ICD‑10 = *International Classification of Diseases, Tenth Revision*; ICPC = International Classification of Primary Care.

Note: Positive predictive value was calculated as PPV = true positives / (true positives + false positives + nonevaluable cases). Results are presented as PPVs (%) and their 95% CIs.
